# Commentary: Type 2 diabetes self-management: spirituality, coping and responsibility

**DOI:** 10.1177/17449871211030595

**Published:** 2021-12-21

**Authors:** Stella F. Bosun-Arije

**Affiliations:** Senior Lecturer, Department of Nursing, Manchester Metropolitan University, UK

I read with interest the article entitled ‘Type 2 diabetes self-management: spirituality, coping and responsibility’. In England, the updated clinical guideline by the National Institute for Health and Care Excellence (NICE, 2020) recommends an individualised or person-centred approach to diabetes care. This is because an individualised approach makes it possible for health professionals to prioritise and understand patients’ preferences as well as assess patients’ needs. In light of optimising person-centred care, this article highlights that understanding a patient’s spirituality is often neglected in the real world of clinical practice. This is particularly disappointing given the vast spiritual diversity that exists in our current world. A world where 1 in 11 adults lives with diabetes (International Diabetes Federation, 2019).

Type 2 diabetes mellitus (T2DM) is often a preventable type of DM, yet the past decade, however, has seen an increasing prevalence of T2DM and more management challenges (World Health Organization (WHO), 2016). Management challenges often lead to uncontrolled hyperglycaemia and early onset of complications of T2DM such as retinopathy, nephropathy and neuropathy). These complications can potentially lead to health-limiting and life-threatening conditions (Zhang et al., 2020). Whereas, there is an abundance of evidence that self-care plays a cogent role in the early identifications and effective management of complications (Lainscak et al., 2011; Minet et al., 2010; Shrivastava et al., 2013).

In light of the above, this paper provides an insight into the role of spirituality in optimising patient self-care in the 21st century. Self-care is crucial for a patient with diabetes to develop autonomy in making informed health choices and lifestyle modifications that can heighten positive health outcomes. This research rationale is theoretically driven and it provides a convincing justification for health professionals to reflect on the role that spirituality plays in dictating coping styles or informing patients’ sense of responsibility to engage in self-care activities.

Given qualitative data analysis is often critiqued to be less transparent, an unequivocal, step-by-step approach to data analysis could be presented with computer software such as the NVivo. Although the findings of the research are easy to read and understand however, a graphical presentation of findings may improve the understanding of non-clinical practitioners when they read this paper.

Strategies for person-centred T2DM management can be effective when collaboration and partnership are integrated into patient care. Patients, their relations, healthcare professionals, caregivers within healthcare settings, community, local and national governments should work with health policymakers to ensure effective implementation of strategies in favour of the promotion of self-care management for patients diagnosed with diabetes.

In summary, this paper has contributed to a critical part of diabetes care and I recommend that health professionals should continue to seek practical ways to tangibly apply these research findings in real-world clinical settings. The findings can be discussed and shared in different forums such as workshops, seminars and webinars. It is my great pleasure to have reviewed this article and I hope health professionals continue to seek a deeper understanding of various factors that can facilitate the promotion of diabetes self-care management.
